# A Novel Stem Cell Model to Study Preeclampsia Endothelial Dysfunction

**DOI:** 10.1007/s43032-024-01590-z

**Published:** 2024-08-23

**Authors:** Yanming Wu, Tianyanxin Sun, Pedro Medina, Purnima Narasimhan, David K. Stevenson, Frauke Von Versen-Höynck, Jennifer Armstrong, Joseph C. Wu, Nazish Sayed, Virginia D. Winn

**Affiliations:** 1 Department of Obstetrics and Gynecology, Stanford University School of Medicine, Stanford, CA USA; 2 Stanford Cardiovascular Institute, Stanford University School of Medicine, Stanford, CA USA; 3 Department of Pediatrics, Division of Neonatal and Developmental Medicine, Stanford University School of Medicine, Stanford, CA USA; 4 Department of Obstetrics, Gynecology and Reproductive Sciences, Hannover Medical School, Hannover, Germany; 5 Department of Pediatrics, Section of Neurology and Department of Obstetrics and Gynecology, Division of Basic Reproductive Sciences, University of Colorado Anschutz Medical Campus, Aurora, CO USA; 6 Division of Cardiovascular Medicine, Stanford University School of Medicine, Stanford, CA USA; 7 Division of Vascular Surgery, Department of Surgery, Stanford University School of Medicine, Stanford, CA USA

**Keywords:** Preeclampsia, Pregnancy, Hypertension, Endothelial dysfunction, Induced pluripotent stem cells (iPSCs)

## Abstract

Preeclampsia is a common pregnancy complication affecting 5% to 7% of all pregnancies worldwide annually. While the pathogenesis is not fully understood, maternal endothelium dysfunction is thought to be a central component to preeclampsia development. Studies to dissect maternal endothelial dysfunction, particularly on a patient-specific basis, are hampered by limited access to systemic primary endothelial cells (ECs). The objective of this study was to establish a replenishable, patient-specific in vitro EC model to allow robust mechanistic studies to dissect endothelial dysfunction in preeclampsia. Induced pluripotent stem cells (iPSCs) from three women with a history of normotensive pregnancies were differentiated into ECs. The established ECs were exposed to pooled sera from normotensive pregnancies, preeclamptic pregnancies, normotensive postpartum for non-pregnant comparison and controls. Endothelial functions including nitric oxide (NO) release, cell migration, tube formation and viability were evaluated. Levels of NO release were significantly lower after incubation with preeclamptic sera compared to the fetal bovine serum (FBS) control, and normotensive and non-pregnant (postpartum) sera treatments were also lower than FBS but higher than preeclamptic sera treatments. Tube formation and cell migration were also impaired with preeclamptic sera compared to FBS controls. Cell viabilities remained unaffected by any sera treatment. Consistent outcomes were obtained across all three patient-specific lines treated with the same pooled sera. Establishment of patient-derived iPSC-ECs treated with pregnancy sera serves as a novel model to explore the interplay between individual maternal endothelial health and circulating factors that lead to endothelial dysfunction in preeclampsia.

**Supplementary Information:**

The online version contains supplementary material available at 10.1007/s43032-024-01590-z.

## Introduction

Preeclampsia is a common pregnancy-specific complication affecting 5% to 7% of all pregnancies worldwide annually [1, 2]. Preeclampsia is defined by new onset of hypertension and significant end-organ dysfunction with or without proteinuria after 20 weeks of gestation [3, 4]. Despite increased efforts in developing predictive biomarkers and prophylactic treatments [5], results have been inconsistent [6–9], and delivery remains the only definitive management for preeclampsia [10]. This could be attributed to the fact that preeclampsia is a syndrome with different pathways to its development, which is supported by an emerging understanding of subtypes at the molecular level.

Maternal endothelial dysfunction is considered as a key pathophysiologic contributor to preeclampsia, underlying most clinical manifestations, including hypertension and proteinuria [11, 12]. Impaired placentation leading to uteroplacental ischemia and subsequent release of factors into the maternal circulation are proposed to trigger systemic endothelial responses [12–14]. The role of placenta-derived factors is further supported by evidence of an imbalance of pro-angiogenic/anti-angiogenic factors, increased biomarkers for pro-inflammatory cytokines, lipid peroxidation, and oxidative stress detected in the maternal circulation system in preeclamptic pregnancies [11, 12, 15, 16]. Targeted directly by placenta-derived factors, endothelial cells (ECs) can be activated through multiple pathways, resulting in widespread endothelial dysfunction including, but not limited to, impaired vasodilatory capacity, vascular permeability, and an increased anti-angiogenic state [11, 17, 18]. Thus, both maternal endothelium and placenta-derived factors play a critical role in the mechanism of developing preeclampsia.

Impaired placentation may not be the only triggering factor, since impaired spiral artery vascular invasion is not seen in all preeclamptic cases [19], and patients can also develop preeclampsia postpartum [20, 21]. Women with chronic diseases, such as hypertension, chronic kidney diseases, and diabetes mellitus, which are related to impaired endothelial condition, are found to be more susceptible to preeclampsia [22, 23]. In addition, it is recognized that preeclampsia has a genetic predisposition in a maternal dominant manner, and overall heritability is estimated at ~ 55% [24, 25]. Numerous common variants at different maternal gene loci have been associated with increased risk of preeclampsia [26–28]. However, advances are hampered by the heterogeneity of the disease as no single cause can account for all cases of preeclampsia. It is understandable that a complex mix of factors, including maternal genetic factors, preexisting endothelial conditions prior to pregnancy, and circulating factors derived from an impaired placenta, serve as key components in the development of PE.

Thus, the objective of this study is to establish a patient-specific EC model to recapitulate maternal endothelial response to pregnancy sera in a controllable manner. Elucidating the mechanisms of endothelial dysfunction can provide a foundation for the development of biomarkers and targeted therapeutic approaches, and will also enable direct testing of specific genetic variants that may contribute to endothelial dysfunction in preeclampsia. The ultimate goal will be to leverage this innovative patient-specific EC model to advance mechanistic studies examining both the placenta-derived circulating factors and the maternal genetic contributions to PE. Through this research, we aim to contribute to the development of improved diagnostic tools, targeted therapies, and preventive strategies that will significantly impact the management of PE and improve outcomes for both mothers and babies.

## Materials and Methods

### Blood Sample Collection for Pooled Pregnancy Blood Sera

The study participants provided written informed consent under Institutional Review Board (IRB)-approved protocols (#34745, #32773 and COMIRB 09–1107) for blood collection for analyses and banking. Maternal venous blood samples were collected in red top tubes (BD Vacutainer®, Bectin, Dickinson and Co, Franklin Lakes, NJ, USA) from normotensive pregnancies (N, *n* = 13), preeclamptic pregnancies (PE, *n* = 12), and at 6 + weeks postpartum from normotensive pregnancies (PP-N, *n* = 9) as a non-pregnant comparison. PE is defined as new onset of hypertension (systolic blood pressure ≥ 140 mmHg and/or diastolic blood pressure ≥ 90 mmHg diastolic) after 20 weeks of pregnancy plus proteinuria (> 300 mg/24 h, or urine protein to creatinine ratio of > 0.3, or 1 + on urine dipstick) and/or other end organ dysfunction [3]. All participants had singleton pregnancies and did not have any histories of cardiovascular diseases.

Collected venous blood was allowed to clot at room temperature and then centrifuged at 2000 g for 10 min at 4 °C. The supernatants of the centrifuged sera were biobanked at -80 °C. For the endothelial experiments, sera aliquots from each subject were thawed on ice, combined into a single pooled sample and then aliquoted (100µL) and frozen again at -80 °C until use for each experiment. Samples were selected to cover both preterm and term pregnancies. This provided common sera across all experiments with the same freeze thaw history and represented the spectrum of PE. Clinical demographic variables were recorded and provided in Supplemental Table 1.

### Generation of Induced Pluripotent Stem Cells (iPSCs)

The study participants provided written informed consent for isolation of human peripheral blood mononuclear cells (PBMCs) and generation of iPSCs under Stanford University IRB (#62122) and Stem Cell Research Oversight (SCRO) panel (#856). Peripheral blood from women with history of normotensive pregnancies (Supplemental Table 2) were collected and centrifuged at 1700 g for 20 min at room temperature in Cellular Preparation Tubes (CPT, Bectin, Dickinson and Co). PBMCs were separated according to published protocols [29]. PBMCs were reprogrammed to iPSCs by the Stanford Cardiovascular Institute Biobank [30]. The karyotype information of each established iPSC line was matched to the original patient cells using single nucleotide polymorphism (SNP) analysis. Pluripotency was confirmed using the TaqMan hPSC Scorecard™ Assay.

### Differentiation of Patient-Specific iPSCs Into ECs

The differentiation of ECs from iPSCs followed the protocol previously described [31] with minor modifications. Endothelial differentiation was started from iPSCs with cell passages between fifteen and twenty. Briefly, patient-specific iPSCs were incubated in StemMACS™ iPS-Brew XF and passaged at least twice before differentiation was initiated. iPSCs were first induced into the mesoderm cells by activation of the WNT signaling pathway using 6 µM of CHIR99021 (Selleck Chemicals LLC, Houston, TX, USA) for 2 days, and then followed by 2 µM of CHIR99021 in RPMI medium supplemented with B-27 minus insulin (Gibco) for another 2 days. Cells were further differentiated into the endothelial lineage with the addition of 50 ng/mL of vascular endothelial growth factor (VEGF) (PeproTech, Rocky Hill, NJ, USA) and 20 ng/mL of Fibroblast Growth Factor (FGF) (PeproTech) in EGM-2 media (Lonza, Basal, Switzerland) for eight days. Media were changed every other day. On the twelfth day of differentiation, ECs were purified via magnetic-activated cell sorting (MACS) incubated with CD144 MicroBeads (Miltenyl Biotec, San Jose, CA, USA). iPSC-ECs were labeled as passage zero after MACS. The differentiated ECs were cultured in EGM-2 medium supplemented with 10 µM of SB431542 (Selleck Chemicals) and 5 ng/mL of VEGF on the plates precoated with 0.2% gelatin (Sigma-Aldrich, St. Louis, MO, USA) until passage one and then were counted and frozen for further use. The established ECs were labeled as EC_1, EC_2, and EC_3 to indicate different subject origin. iPSC-ECs at passage three were used for the functional assays.

### RNA Extraction and Real-Time PCR

Total RNA of from the three patient-derived iPSC-ECs at passage three were extracted using RNeasy Midi Kit (Qiagen, Ann Arbor, MI, USA) and were quantified by using a NanoDrop Spectrophotometers. 1 μg of RNA was reversed transcribed to cDNA using iScript cDNA Synthesis Kit (Bio-Rad Laboratories, Hercules, CA, USA) in a 20 μL reaction volume. Primers were synthesized by Stanford Protein and Nucleic Acid Facility as follows: Von Willebrand Factor (vWF) forward, 5’-GTACAGCTTTGCGGGATACT-3’; vWF reverse, 5’-GCTCACTCTCTTGCCATTCT-3’; CD31 forward, 5’-CCGATGTCAAGCTAGGATCATT-3’; CD31 reverse, 5’-GATGTGGAACTTGGGTGTAGAG-3’; Notch 1 forward, 5’-ATGTGTTCTCGGAGTGTGTATG-3’; Notch 1 reverse, 5’-AGGGACCAAGAACTTGTATAACC-3’; GAPDH forward, 5’-TCGGAGTCAACGGATTTGGT-3’; GAPDH reverse, 5’-TTCCCGTTCTCAGCCTTGAC-3’. Real-time PCR was performed using PowerUp™ SYBR™ Green Master Mix (Applied Biosystems, Waltham, MA, USA) according to the manufacturer’s instruction. The real-time PCR cycling condition was 2 min at 50 °C for uracil DNA glycosylase (UDG) activation, 2 min at 95 °C for Dual-Lock DNA polymerase incubation, followed by 40 cycles of 15 s at 95 °C and 1 min at 60 °C. The amplification specificity was confirmed by the melting curve of each PCR product. Levels of mRNA expression were analyzed using the 2^−ΔCT^ method by normalizing to GAPDH.

### Immunofluorescence Staining of ECs

To characterize the purity of the differentiated ECs from patient-derived iPSCs, immunofluorescence staining of ECs markers was performed. Cells were cultured in EGM-2 media on chamber slides (BD Falcon, BD Biosciences, San Jose, CA, USA) precoated with 0.2% gelatin until 80% confluence was obtained. Cells were fixed with 4% paraformaldehyde solution for 10 min at room temperature. For staining, cells were first permeabilized with 0.3% TritonX-100, blocked by 2% horse serum (Vector Laboratories, Newark, CA, USA) for one hour, and then probed with 0.5% horse serum containing rabbit anti-vWF (Abcam, Boston, MA, USA, 1:200 dilution), mouse anti-CD31 (Thermo Scientific, Ann Arbor, MI, USA, 1:100 dilution), Mouse anti-Notch 1 (Thermo Scientific, 1:100 dilution) and Mouse anti-eNOS (BD Biosciences, 1:100 dilution) overnight at 4 °C. The cells were washed with 0.1% bovine serum albumin (BSA) twice and incubated with Alexa Fluor™ 488-conjugated donkey anti-rabbit/mouse IgG (Invitrogen, Waltham, MA, USA, 1:200 dilution) in 0.5% horse serum for 2 h at room temperature. Cells were washed with 0.1% BSA once and mounted in DAPI mounting medium (Vector Laboratories) for nuclear staining. Images were visualized using a Leica DM IL LED inverted microscope (Stanford University, Palo Alto, CA) and processed with Leica Application Suite X software.

### Functional Assays of ECs

For cellular viability assays, ECs were seeded in 96-well plates (5 × 10^3^ cells/well) and cultured in EGM-2 media supplemented with 5% pooled banked sera from N, PE, and PP-N pregnancies. Basal media with or without 5% fetal bovine sera (FBS) served as additional controls. After 24 h, cellular viabilities were evaluated using MTS Cell Proliferation Assay Kit (Abcam).

For nitric oxide (NO) release and cell migration assays, ECs were seeded in ImageLock 96-well plates (2.5 × 10^4^ cells/well) and cultured in EGM-2 media supplemented with 5% FBS or 5% of pooled banked sera as indicated above. After 24 h, supernatants were freshly collected to measure levels of total NO release (nitrate and nitrite) using Nitrate/Nitrite Fluorometric Assay Kit (Cayman Chemical, Ann Arbor, MI, USA). A nitrate standard curve was generated to quantitate the sample NO concentrations measured at excitation/emission wavelengths of 375/417 nm. Meanwhile, a linear wound was generated across the attached monolayer cells using an IncuCyte Woundmaker. Cells continued to be maintained in conditioned media. After another 24 h, cell migration was visualized using an IncuCyte camera and relative wound densities were analyzed using the IncuCyte Live-Cell Analysis System.

For tube formation, ECs were pre-treated with 5% FBS or 5% of pooled banked sera for 24 h, and then seeded in 96-well plates (2.5 × 10^4^ cells/well) pre-coated with 50 μL of Matrigel (Corning, Bedford, MA, USA). Cells were maintained in conditioned media for another 8 h, and tube formation was imaged by the IncuCyte camera. The number of meshes and total segments length were quantified by ImageJ (NIH, Bethesda, MD, USA).

### Statistical Analyses

All data are presented as mean ± standard error (SEM) or mean ± standard deviation (SD) as indicated. All experiments were conducted in triplicate or quadruplicate for all three patient-derived ECs. Gene expressions for comparison of differentiated EC lines with iPSCs were analyzed by unpaired Student’s t test with Welch’s correction. For comparison of cell response to sera treatments, multiple group comparisons were performed by one-way ANOVAs followed by Tukey tests. Significant differences were defined as *P* < 0.05.

## Results

### Demographics

Banked sera were available from three cohort studies where participants consented to future use of their blood. The characteristics of patients’ sera used are shown in Supplemental Table 1. Biobanked sera was pooled from 13 women with normotensive pregnancy (N), 12 women with preeclamptic pregnancy (PE), and 9 women who were postpartum after normotensive pregnancy (PP-N). Of the PE women, 58.3% presented with early-onset preeclampsia (before 34 weeks of gestational age), and 83.3% were diagnosed with severe preeclampsia as defined by ACOG [2]. To mitigate the effects of gestational progression, the gestational days at sera collection were comparable between the N and PE groups. Sera samples from the PP-N group were collected within 45 to 77 days postpartum (61.6 ± 11.6), serving as a non-pregnancy comparison. Consistent with the actual gestational age, fetal weight in both the N and PE groups was significantly lower than in the PP-N group, as most of the patients in these groups had preterm deliveries. Maternal age of the available PP-N group was higher than both N and PE group. Body mass index (BMI) was significantly higher in the PE group compared to either the N or PP-N group. There were no statistical differences observed in patients’ ethnicity, fetal sex, parity, and gravity across all the groups.

### Characterization of Patient-Derived ECs

**Fig. 1:**
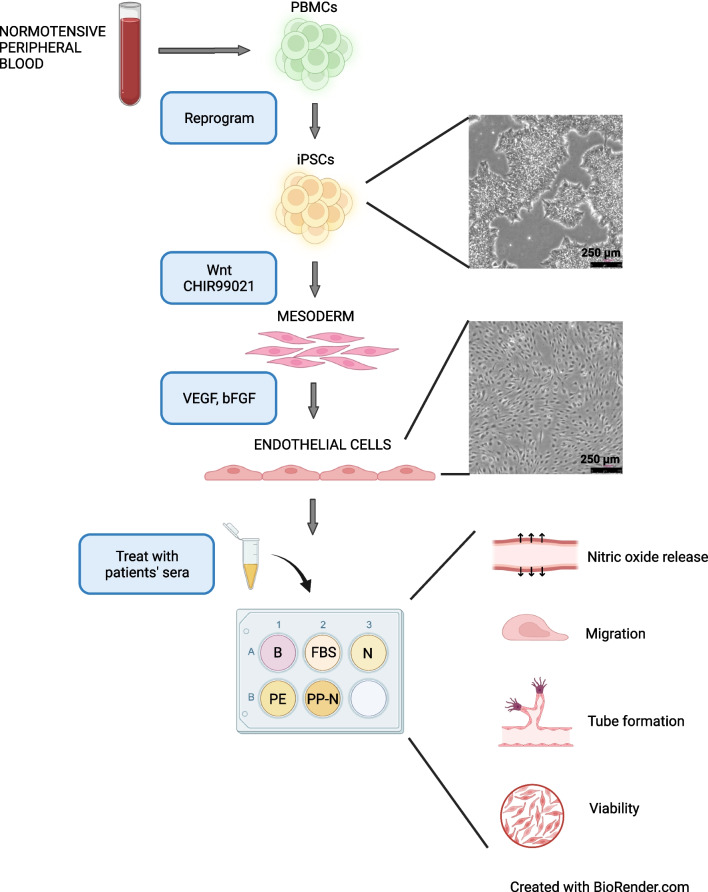
Workflow of generation of patient-derived iPSC-endothelial cells (ECs) and assessment for endothelial functions. Peripheral blood mononuclear cells (PBMCs) were isolated from normotensive maternal blood and were reprogrammed into induced pluripotent stem cells (iPSCs). Then, iPSCs were induced into the mesoderm cells by activating WNT signaling and were further differentiated into ECs with addition of growth factors. Cell morphologies of iPSCs and differentiated ECs are shown. The differentiated ECs were pretreated with basal media (B) or 5% of fetal bovine sera (FBS), pooled patients’ sera from normotensive pregnancy (N), preeclampsia (PE) and normotensive 6 + weeks postpartum (PP-N), respectively, with functional assays analyzed subsequently

**Fig. 2:**
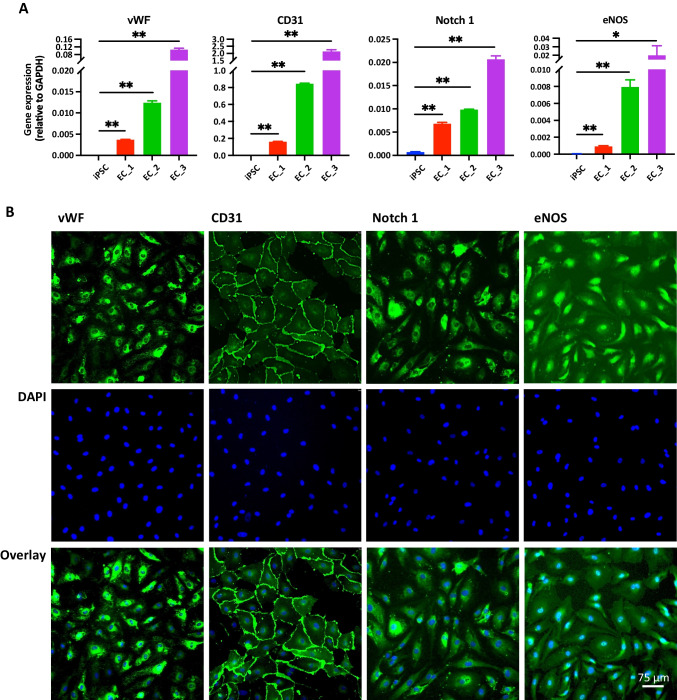
Characterization of patient-derived ECs. Successful ECs differentiation was indicated by elevation of mRNA levels and positive immunofluorescence staining of specific EC markers. **A** Gene expression of vWF, CD31, Notch 1 and eNOS in iPSCs compared to the three established ECs. **B** Immunofluorescence staining of vWF, CD31, Notch 1 and eNOS in one representative EC line. The cells were counterstained with DAPI as a nuclear marker. vWF, von Willebrand factor; eNOS, endothelial NOS; ECs, endothelial cells; DAPI, 4’,6-diamidino-2-phenylindole. Quantitative data are expressed as means ± SEM (*n* = 3). *P < 0.05, **P < 0.001

The differentiated ECs were characterized by cell morphology, gene expression, and immunofluorescence staining with specific endothelial markers [32, 33]. Once the ECs reached confluence after MAC sorting, microscopic images under the bright field showed the typical cobblestone-like appearance in monolayer pattern (Fig. 1). The purities of all three patient-derived EC lines were verified by dramatic upregulation of vWF, CD31, Notch 1 and eNOS gene expression compared to the parent iPSCs (Fig. 2A). As expected, iPSCs showed no amplification of vWF, CD31 and eNOS, and slight expressions of Notch 1. Significant protein expressions were also visualized in ECs by immunofluorescence staining (Fig. 2B).

### Impaired Endothelial Cell Function by Sera from Preeclampsia Patients

To investigate how patient-derived ECs respond to preeclamptic sera compared to normotensive pregnancy and non-pregnant control sera, endothelial functional assays including cell viability, NO release, cell migration, and tube formation involved in angiogenesis were explored in the three established ECs [34] (Fig. 1).

**Fig. 3:**
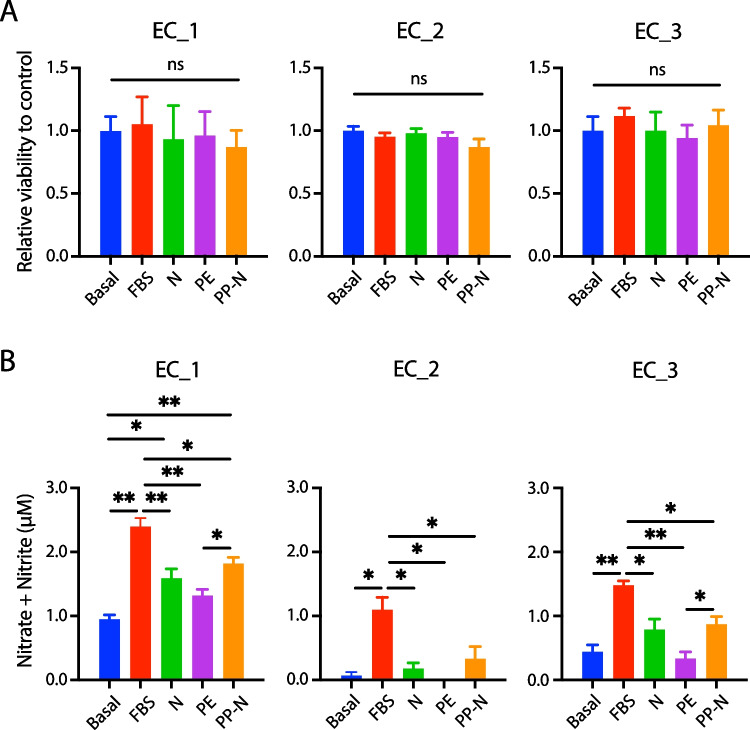
Viability and nitric oxide (NO) release after patients’ sera treatment in three iPSC-EC lines. ECs were differentiated from three individuals with normotensive pregnancy. ECs were pretreated with basal media or 5% of sera (FBS, PE, N, and PP-N) before preforming the functional assays. **A** Cellular viabilities were not affected among all the treatments. **B** Levels of NO release upon preeclamptic sera treatment were significantly reduced when compared with FBS treatment. Quantitative data are expressed as means ± SEM (*n* = 3–4). *P < 0.05, **P < 0.001

All three patient-derived ECs demonstrated similar cell response to pooled banked sera. No significant difference in cell viabilities was observed upon different treatments across the three EC lines (Fig. 3A). Since vascular endothelium play a critical role in regulating vascular tone via the synthesis and release of vasodilator substances, the ability to produce NO was evaluated as a surrogate of endothelial function [35]. Concentrations of nitrate (NO_3_^−^) and nitrite (NO_2_^−^) were quantified as total amount of NO products released into the media. Among all the treatments, NO levels were dramatically reduced by PE sera, with comparable or even lower levels than the non-treatment basal conditions. Production of NO after incubation with N or PP-N sera was also lower than FBS treatment, but higher than PE sera treatment (Fig. 3B).

**Fig. 4:**
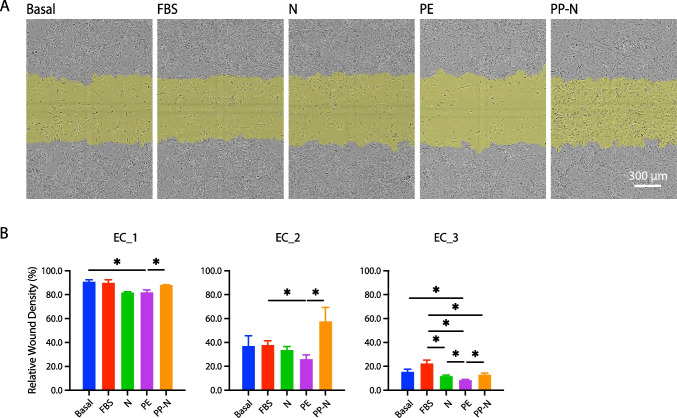
Cell migration in response to pooled patients’ sera. iPSC-ECs were pretreated with basal media or 5% of sera before performing wound healing assay. **A** Representative pictures demonstrated cell migration under each treatment condition at 24 h. The shaded areas in yellow represent the initial wound scratch. **B** Quantitative data are expressed as mean ± SEM (*n* = 4). *P < 0.05

**Fig. 5:**
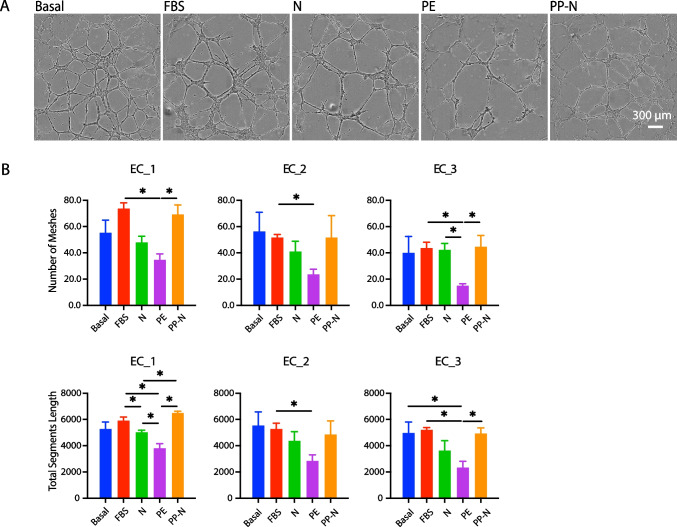
Tube formation in response to pooled patients’ sera. iPSC-ECs were pretreated with basal media or 5% of sera before splitting and plating on Matrigel with continuous treatment. **A** Representative pictures demonstrated EC tube formation under each treatment condition at 8 h. **B** Quantitative data are expressed as mean ± SEM (*n* = 3). *P < 0.05

EC functions involved in angiogenesis were assessed by the classic standard EC assays of cell migration (wound healing assay) and tube formation. As shown in Figs. 4 and 5, the overall ability to migrate and form capillary-like structures varied by the different EC lines. EC_1 almost healed the wound after 24 h and formed the greatest number of meshes and total segments length after 8 h, followed by EC_2 and EC_3. Cell response to pooled banked sera demonstrated similar pattern across all three EC lines. Representative figures of cell migration (Fig. 4A) and tube formation (Fig. 5A) after incubation with different sera in EC_2 was demonstrated. EC migration and tube formation were significantly inhibited by PE sera treatment compared to FBS and/or basal conditions. The levels of NO release, rate of cell migration, and abilities of tube formation were notably upregulated by PP-N sera treatment compared to PE sera treatment in all three EC lines (Figs. 4B and 5B).

## Discussion

Maternal endothelial dysfunction has been widely accepted as a crucial contributor in the development of preeclampsia [1, 11, 13, 36–38]. However, studies to understand the mechanistic aspects of maternal endothelial dysfunction are largely hampered by the limited access to systemic primary ECs. In this study, we established a novel iPSC-ECs model to study maternal endothelial responses in a patient-specific manner. The differentiation of ECs from iPSCs bypasses the difficulties in accessing primary ECs and provides a replenishable resource for maternal ECs. In accordance with the common features of ECs, our three iPSC-EC lines demonstrated cobblestone-like shape and significantly expressed well-recognized EC markers (eg, vWF, CD31, Notch 1, eNOS) both at the transcriptional and protein levels, indicating the structural similarities between our iPSC-ECs and established EC lines [39, 40].

Maternal endothelial functions, assessed through NO release, migration, and tube formation, exhibited varied responses to the various pooled sera, suggesting the presence of distinct circulating factors in patients’ sera that differentially influenced maternal endothelial function. Notably, endothelial functions were suppressed in response to PE sera compared to FBS control and/or N sera, recapitulating the maternal endothelial dysfunction in preeclampsia. Importantly, these functional impairments were not attributed to cell death, as no significant difference in cell viability was observed among all the treatments. Enhanced endothelial functions were also observed in response to PP-N sera compared to PE sera. However, whether this observation will be replicated with postpartum sera from preeclampsia, or whether a more blunted return to baseline will be seen, is of great interest. Although similar patterns of endothelial response to sera were found, variabilities were also observed among different patient-derived ECs, which is most likely attributed to the different genetic background of each individual. Studies have shown that genetic differences between individual donors could cause transcriptional variation among iPSC lines, whereas other factors such as the reprogramming strategy or cell type of origin has a minimal role in iPSCs’ heterogeneity [41, 42]. Moreover, a comprehensive characterization of differentiated iPSC derivatives to understand the inter- and intra-patient genetic heterogeneity showed that a greater inter-patient variation exist that might be contributing to the observed phenotypic variation between the different donors [43]. The noted variabilities in our study may explain the various clinical manifestations seen in patients, suggesting that the genetic background of the pregnant person may contribute to not only preeclampsia development but also the clinical presentation.

In this study, we did not attempt to determine which circulating factors in the sera triggered the endothelial dysfunction, nor what accounted for the differential response by the different patient-derived ECs. Further studies are anticipated to examine whether iPSC-ECs from preeclamptic pregnancies will demonstrate similar or diverse responses to the same pooled sera. With advancement of metabolomic and extracellular vesicle analysis along with genome wide sequencing and CRISPR-based gene editing techniques, we are now poised to explore the underlying mechanisms behind endothelial dysfunction in preeclampsia from the perspective of both the placental circulating factors and the maternal genetic background. Elucidating the intricate interplay between these factors and maternal endothelial cells will contribute to a better understanding of the molecular and genetic basis of preeclamptic endothelial dysfunction. Importantly, our iPSC-ECs endothelial dysfunction platform has promising clinical applications, and can be used to assess ECs response to pharmacologic agents [39, 44], thereby guiding personalized treatment strategies.

## Conclusions

The establishment of patient-specific iPSC-ECs provides a novel in vitro cell model to advance mechanistic studies of preeclampsia. This fills an important gap in the field by offering a novel approach to circumvent the challenges in accessing primary endothelial cells and creating a replenishable cell source. In agreement with our current hypothesis, maternal endothelial dysfunctions were observed in response to PE sera, indicating the validity of the cell model. This model provides the ability for future investigations into the specific contributions of maternal serum constituents to preeclampsia pathogenesis, particularly focusing on placenta-derived factors [45, 46]. We are now poised to explore specific molecular targets, determine endothelial gene expression signatures related to PE endothelial dysfunction, directly examine genetic susceptibility, and use the model for potential therapeutic screening. Collectively, these strategies will help expand our knowledge about the pathogenesis of preeclampsia, and guide personalized medicine approaches for early prediction, prevention, and treatment of preeclampsia.

## Supplementary Information

Below is the link to the electronic supplementary material.

Supplementary file1 (DOCX 17.1 KB)

## Data Availability

All data generated is included in this publication.
