# Supplementary material for: A Novel Stem Cell Model to Study Preeclampsia Endothelial Dysfunction
Source: Reprod Sci. 2024 Aug 23;31(10):2993–3003. doi: 10.1007/s43032-024-01590-z (PMC11438721; doi:10.1007/s43032-024-01590-z)
Supplement: Supplementary file 1 — Supplementary file1 (DOCX 17.1 KB) [file 43032_2024_1590_MOESM1_ESM.docx]

**Supplemental table 1:**

**Patient demographics**

| **Demographic** | **N (n=13)** | **PE (n=12)** | **PP-N (n=9)** |
| --- | --- | --- | --- |
| Maternal age, (y) | 28.5 ± 7.6 | 27.4 ± 6.4 | 36.9 ± 3.4^a^ |
| Obstetric history before enrollment |  |  |  |
| Gravity | 1.7 ± 0.4^b^ | 1.9 ± 0.6^b^ | 1.2 ± 0.3 |
| Parity | 0.4 ± 0.2^b^ | 0.6 ± 0.3^b^ | 0.2 ± 0.2 |
| Ethnicity, n (%) |  |  |  |
| White, non-Hispanic | 8 (61.5%) | 6 (50.0%) | 5 (55.6%) |
| White, Hispanic | 2 (15.4%) | 4 (33.3%) | 1 (11.1%) |
| Asian | 2 (15.4%) | - | 3 (33.3%) |
| Black | - | 1 (8.3%) | - |
| Unknown | 1 (7.7%) | 1 (8.3%) | - |
| BMI at presentation, (kg/m^2^) | 27.3 ± 4.3 | 33.6 ± 5.3^a^ | 24.9 ± 2.8 |
| Unknown, n (%) | 3 (23.1%) | - | - |
| Gestational days |  |  |  |
| At sera collection | 223.7 ± 28.6 | 217.7 ± 28.3 | - |
| At delivery | 232.6 ± 32.9 | 225.5 ±19.8 | 273.0 ± 13.7^a^ |
| Fetal weight, (g) | 2123 ± 955.9 | 1458 ± 571.6 | 3143 ± 544.1^a^ |
| Fetal gender, n (%) |  |  |  |
| Male | 7 (53.8%) | 5 (41.7%) | 4 (44.4%) |
| Female | 6 (46.2%) | 6 (50.0%) | 5 (55.6%) |
| Undisclosed | - | 1 (8.3%) | - |

The data for each group are expressed as mean ± SD. One-way ANOVA followed by Tukey’s multiple comparison test or Student’s t test were performed for statistical comparisons between the groups.

GA, gestational age; PE, preeclampsia; PP-N, postpartum from normotensive pregnancy; BMI: body mass index.

^a^ A value of *P* < 0.05 was considered statistically significant compared to the other groups.

^b^ One data point was missing.

**Supplemental table 2:**

**Clinical and iPSC Line Information**

| **EC Line** | **EC_1** | **EC_2** | **EC_3** |
| --- | --- | --- | --- |
| Pregnancy History | G1P1  no HDP | G2P2  no HDP | G4P4  no HDP |
| Gender | Female | Female | Female |
| Race | White | Other | White |
| Ethnicity | Not Hispanic or Latino | Hispanic or Latino | Not Hispanic or Latino |
| iPSC Line | SCVI823 | SCVI854 | SCVI2041 |

GxPx: Gravity and Parity

HDP: hypertensive disease of pregnancy

SCVI: Stanford Cardiovascular Institute
